# The diachronic trend of female and male stature in Milan over 2000 years

**DOI:** 10.1038/s41598-023-28406-5

**Published:** 2023-02-23

**Authors:** Lucie Biehler-Gomez, Beatrice del Bo, Daniele Petrosino, Paolo Morandini, Mirko Mattia, Luca Palazzolo, Uliano Guerrini, Cristina Cattaneo

**Affiliations:** 1 LABANOF, Laboratorio di Antropologia e Odontologia Forense, Sezione di Medicina Legale, Dipartimento di Scienze Biomediche per la Salute, Università degli Studi di Milano, Via Mangiagalli 37, 20133 Milan, Italy; 2 Dipartimento di Studi Storici, Università degli Studi di Milano, Milan, Italy; 3 Laboratorio di Biochimica e Biofisica Computazionale, Dipartimento di Scienze Farmacologiche e Biomolecolari, Università degli Studi di Milano, Milan, Italy

**Keywords:** Anthropology, Archaeology

## Abstract

Stature is a biological trait directly determined by the interaction of genetic and environmental components. As such, it is often evaluated as an indicator for the reconstruction of skeletal biological profiles, past health, and social dynamics of human populations. Based on the analysis of 549 skeletons from the CAL (*Collezione Antropologica LABANOF*), a study of the diachronic trend of male and female adult stature in Milan (Italy) is being proposed here, covering a time span of about 2000 years, ranging from the Roman era to present-days. The skeletons, from necropolises dedicated to the less wealthy classes of Milanese society, were assigned to one of following five historical periods: Roman Era (first–fifth centuries AD), Early Middle Ages (sixth–tenth centuries AD), Late Middle Ages (eleventh–fifteenth centuries AD), Modern Era (sixteenth–eighteenth centuries AD) and Contemporary Era (nineteenth–twentieth centuries AD), and their stature was estimated according to the regression formulae of Trotter (1970). The collected data were then subjected to statistical analyses with ANOVA using R software. Although stature values showed an ample standard deviation in all periods, statistical analyses showed that stature did not significantly vary across historical periods in Milan for both sexes. This is one of the rare studies showing no diachronic changes in the trend of stature in Europe.

## Introduction

Stature is a complex trait predominantly determined by genetic factors but with a large environmental component. Although it is 75–90% hereditary determined^1,2^, it also depends on multifactorial causes including fetal and childhood nutrition and health. Because of this interplay of genetic and environmental agents, stature has been used in biological anthropology as a stress marker that can reflect climate adaptation, nutritional conditions, and health status. Indeed, changes in height over time have been related to climate change, socio-economic inequality, demographic cycles, urbanization, and the spread of diseases^3,4^. These environmental factors may alter growth velocity and prevent the individual from reaching its genetic growth potential, making stature a sensitive tool for reconstructing living conditions and microevolutionary trends^1,2,5^.

Diachronic studies on stature have been performed in various regions of the world^6,7^ (among others). In Europe, studies have shown negative secular trends in adult stature related to climate change causing cold temperatures, wars and religious conflicts, the stress of industrialization, rapid urbanization, urban overcrowding, increased population densities, poor hygiene, food shortages, unemployment, inequality of incomes, reduced wages, spread of new diseases and epidemics, as well as height recoveries thanks to economic prosperity, climate favorable to crops leading to higher yields in agriculture, increased productivity and the development of technology, political stability, smallpox inoculation, and public health measures and monitoring^3,5,8–16^.

In a similar perspective, this paper presents the diachronic trend of female and male adult stature in a single place, the metropolitan city of Milan (Italy) and over 2000 years, spanning from the Roman era to Contemporary times. To the best of our knowledge, this is the first time that a study on the evolutionary trend of stature is undertaken in such a confined geographical area and over a long period of time. This setting allows the observation of the evolution of stature in a major European city throughout History and reduces geographical and social biases.

## Materials and methods

The present paper is part of an ongoing project on the reconstruction of the life of the people of Milan throughout History^17–19^, based on the skeletons of the CAL (*Collezione Antropologica LABANOF*—Anthropological Collection of the LABANOF). The CAL is an osteological collection counting about 7000 individuals (about 5000 archaeological remains and 2000 contemporary remains from cemeteries), currently under study at the Laboratory of Forensic Anthropology and Odontology (LABANOF) at the University of Milan.

**Figure 1:**
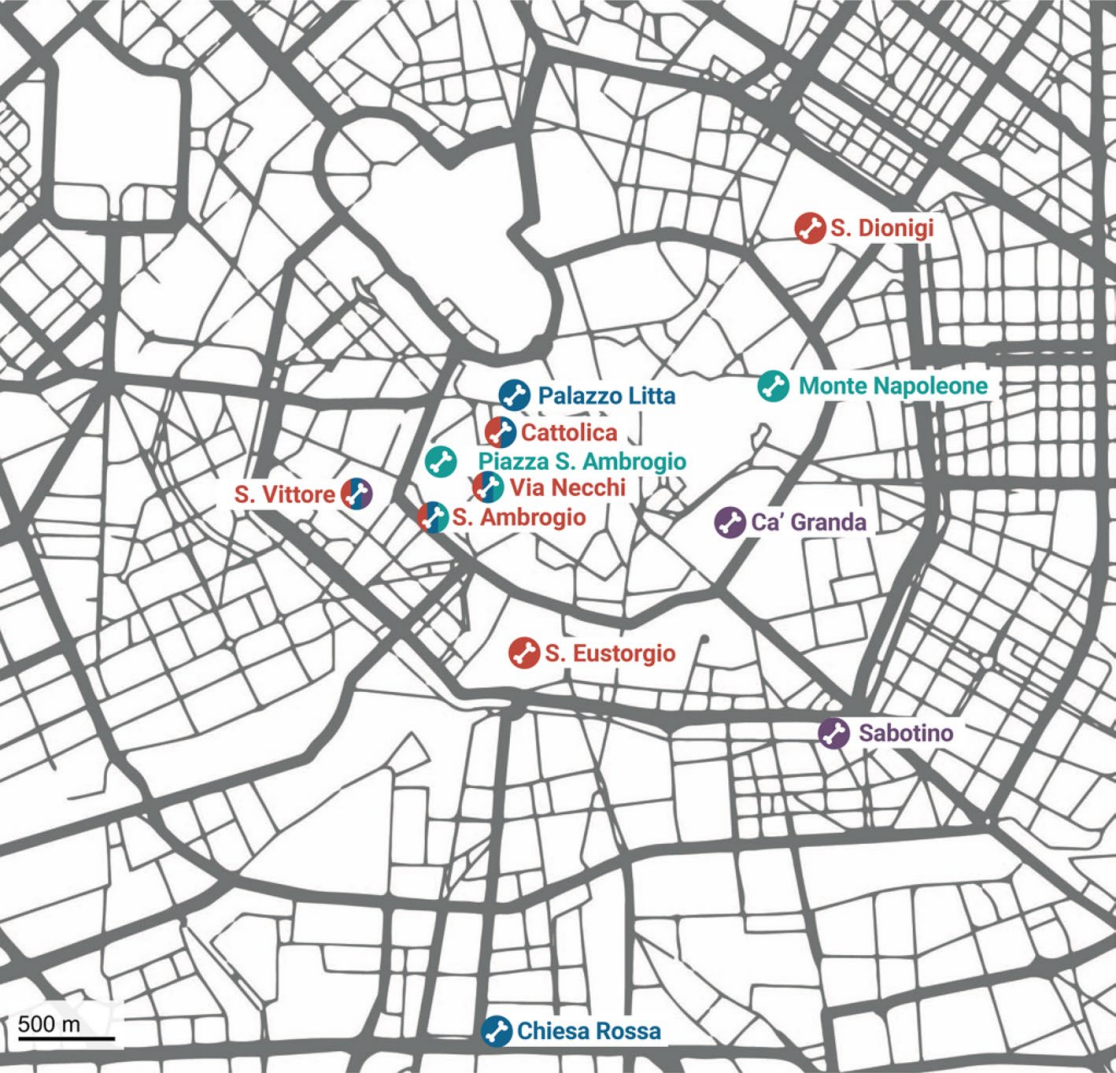
Map of Milan with the different archeological sites selected according to their historical period (red: Roman era, blue: Early Middle Ages, green: Late Middle Ages, purple: Modern era; contemporary cemeteries from the CAL Milano Cemetery Skeletal Collection are located further away from the city center).

For this paper, 549 skeletons of the CAL composed the study sample (Table 1). The skeletons originated from 13 different sites in Milan (Fig. 1): the excavation below the current *Università Cattolica* dated to the Roman era (second–fifth century AD)^20^; that of the roman amphitheater of *Sant’Eustorgio* (fourth century AD); the scientific excavation of the Ambrosian basilica of *San Dionigi* (fifth century AD)^21^; the emergency excavations of *Palazzo Litta* (sixth–tenth century AD), *Chiesa Rossa* (sixth–tenth century AD), *Piazza Sant’Ambrogio* (divided in two areas dated one to the sixth century and the other to the thirteenth–fifteenth century AD)^22^, and a cemetery referable to the church of *Sant’Andrea* in Via* Monte Napoleone* (fifteenth century AD)^23^; the M4 underground metropolitan line vertical excavations at the *Sant’Ambrogio* Basilica (with stratigraphic units spanning from the Roman era—first and second century AD, to the Late Middle Ages—fifteenth century AD) and San Vittore (with phases of burials from the Roman era—third-fourth century AD, to the Modern age—sixteenth and seventeenth century AD, including Tomb 20 from the second half of the fifteenth century which was a burial chamber containing individuals in anatomical connection and commingled remains); the vertical excavation of Via* Necchi* spanning from the Roman era to the Late Middle Ages; the mass grave burials probably due to the Manzoni plague (middle of the seventeenth century AD) from *Viale Sabotino*^24^; the remains of the deceased patients of the Ca’ Granda hospital (in partial or complete anatomical connection—seventeenth century AD)^25^; and the CAL Milano Cemetery Skeletal Collection, a modern and documented osteological collection constituted of unclaimed cemetery individuals who died in the second half of the twentieth century^26^. Dating was performed through stratigraphy, archaeological findings, and radiocarbon analyses of bone samples. For the purpose of this study, the skeletons were attributed to one of five historical periods, divided as follows: Roman era (first–fifth century AD), Early Middle Ages (sixth–tenth century AD), Late Middle Ages (eleventh–fifteenth century AD), Modern era (sixteenth–eighteenth century AD) and Contemporary era (nineteenth–twenty-first century AD). Archaeological data indicate that these sites were necropolises for the poor classes (rarely middle classes) of the Milanese society. The entirety of the sample came from the same urban context, thus allowing a diachronic analysis.

**Table 1:** Details on the study sample.

Period	Site	*n* individuals	*n* females	*n* males
Roman era	Università Cattolica	90	40	50
	Sant’Eustorgio	3	0	3
	San Dionigi	11	7	4
	MM4 Sant’Ambrogio	2	2	0
	Via Necchi	5	2	3
	MM4 San Vittore	10	4	6
	Total	121	56	65
Early Middle Ages	Palazzo Litta	3	0	3
	MM4 Sant’Ambrogio	37	19	18
	Chiesa Rossa	6	2	4
	Università Cattolica	3	1	2
	Piazza Sant’Ambrogio	9	3	6
	MM4 San Vittore	6	2	4
	Via Necchi	42	23	19
	Total	106	50	56
Late Middle Ages	MM4 Sant’Ambrogio	48	22	26
	Via Monte Napoleone	38	23	15
	Via Necchi	14	5	9
	Piazza Sant’Ambrogio	2	0	2
	Total	102	50	52
Modern era	Viale Sabotino	61	24	37
	Crypt of the Ca’ Granda	28	17	11
	MM4 San Vittore	11	9	2
	Total	100	50	50
Contemporary era	CAL Milano Cemetery Skeletal Collection	120	60	60
Total		549	266	283

The skeletons composing the study sample were selected based on two criteria: (1) the coxal bones should be fused (about 16 years of age) in order for sex estimation to be reliably performed using Phenice^27^, Klales et al*.*^28^, Walker^29,30^ and Spradley and Jantz^31^; (2) stature estimation could be performed following Trotter^32^. In this study, all stature estimations were undertaken following Trotter^32^, by applying regression formulae to long bone measurements.

A forward stepwise regression was carried out to consider all interactions between sex, historical period, and site, and find the minimum predictive model by comparing each other using ANOVA (Faraway, 2005). Given that some sites were not equally distributed from a statistical point of view (Table 1), they were aggregated to reach statistical homogeneity of population data. R software was used to perform both linear models and ANOVA statistical analyses, via lm() function and anova() functions, respectively. In detail, different multiple linear regressions were carried out, computing the statistical significance of each categorical variable, and growing the complexity of the model, so as to also consider the interaction among variables. With this strategy, we verified for each model statistical differences among categorical data within each variable, as per previous study^33^. In particular, the interaction between sex and period was tested in the model to verify the sexual dimorphism in stature and the possible secular change in stature. The statistical significance was set at a *p* value < 0.05. Mixed models, via lmer() function in R, were also used setting sex as a fixed variable to verify secondary random effects of other variables on stature but no statistical differences were found comparing models using ANOVA.

### Ethical statements

Study of the archaeological remains was approved by virtue of a convention with the *Sopraintendenza Archeologia, Belle Arti e Paesaggio della Lombardia* (i.e., the regional institution of the Italian ministry of cultural heritage) and undertaken according to ethical and scientific principles per said convention. Examination of the anonymized contemporary remains is consented and regulated by article 43 of the Presidential Decree of the Italian Republic (DPR) n.285 of September 10th, 1990, of the National Police Mortuary Regulation and in accordance with the Health Territorial Agency of the city of Milan. Informed consent was not required. All methods were performed in accordance with the Italian law, institutional guidelines and regulations.

**Table 2:** Descriptive statistics of the study sample.

Periods	Females					Males				
	*n*	Mean	Median	Minimum	Maximum	*n*	Mean	Median	Minimum	Maximum
Roman era	56	157.06	156	146.7	177.6	65	168.71	168.5	154.7	184.7
Early Middle Ages	50	158.17	157.8	145	165.5	58	167.95	168	156.3	179.2
Late Middle Ages	50	157.73	157.1	147.4	172.2	52	169.75	169.9	157.6	179.5
Modern era	50	158.69	158.8	145.5	173	50	167.64	167.9	152	179.1
Contemporary era	60	157.46	157.1	143.5	171.1	60	168.67	168.2	156.6	195.4
TOTAL	266	157.79	162.1	143.5	177.6	283	168.55	164.7	152	195.4

## Results

**Figure 2:**
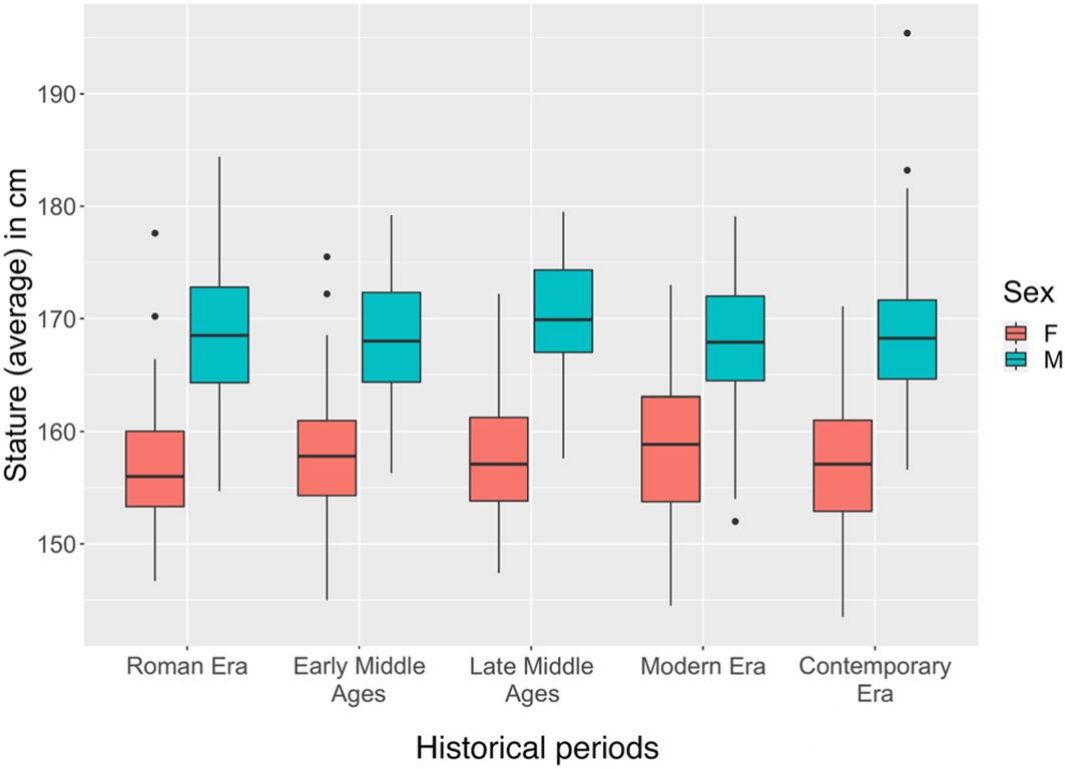
Boxplot visualization of the data according to sex and historical period.

The descriptive statistics of the study sample are presented in Table 2. The sample sizes varied between 50 and 65 individuals per sex and period (the entire dataset in available in the Supplementary Information). The data provided in this paper represent all presently available skeletal individuals from the CAL and excavated from sites in Milan that could be reliably sexed, accurately measured for stature estimation and attributed to a distinct historical period as defined in the Materials and Methods section. As a result of the study, the stature of females appears to vary from 143.5 to 177.6 cm, with a mean of 157.8 cm, whereas the values for males ranged from 152.0 to 195.4 cm, with a mean of 168.5 cm. As observable on Fig. 2, the diachronic trend of mean stature in both males and females remains stable over time. Indeed, statistical analyses showed that historical periods did not significantly influence the variation of the data (*p* = 0.8738), as opposed to sex (*p* < 2e-16) (Table 3). As per the design of the study, bones were used for stature estimation in order of priority according to their standard deviation. Therefore, lower limbs were preferred to upper limbs, and femur and humerus were preferred to tibia/fibula and bones of the forearm, respectively. This preferential selection was dependent upon the state of conservation and preservation of the skeletal remains. Table 4 shows that the most commonly used bones for stature estimation in the present study was the femur, in the overwhelming majority of cases (59.9%), and to a lesser degree, the humerus (18%) and tibia (10.6%). On rare occasions, when state of preservation required it, the bones of the forearm (5.3% each) and fibula (0.9%) were used. Therefore, this study shows that stature does not appear to significantly vary over 2000 years in Milan, for both males and females.

**Table 3:** ANOVA results for the tested linear models.

	ANOVA (Model 1 Vs Model 2)	Pr(> F)
Linear model	Average ~ Sex Vs Average ~ Sex + Period	0.8738
	Average ~ Sex Vs Average ~ Sex + Site_ag	0.8127
	Average ~ Sex Vs Average ~ Sex + Period * Site_ag	0.5492
Mixed model	Average ~ Sex Vs Average ~ Sex + (1|Period)	1
	Average ~ Sex Vs Average ~ Sex + (1|Site_ag)	1

**Table 4:** Frequency of bone use for stature estimation in the sample.

	SD (cm)		Roman era		Early Middle Ages		Late Middle Ages		Modern era		Contemporary era		TOTAL	
	M	F	*n*	%	*n*	%	*n*	%	*n*	%	*n*	%	*n*	%
Femur	3.27	3.72	79	65.3%	63	59.4%	41	40.2%	36	36%	110	91.7%	329	59.9%
Tibia	3.37	3.66	19	15.7%	12	11.3%	11	10.8%	7	7%	9	7.5%	58	10.6%
Fibula	3.29	3.57	2	1.6%	1	0.9%	0	0%	2	2%	0	0%	5	0.9%
Humerus	4.05	4.43	20	16.5%	16	15.1%	31	30.4%	31	31%	1	0.8%	99	18.0%
Radius	4.32	4.30	1	0.8%	11	10.3%	7	6.9%	10	10%	0	0%	29	5.3%
Ulna	4.32	4.30	0	0%	3	2.8%	12	11.8	14	14%	0	0%	29	5.3%

## Discussion

For the first time, the diachronic trend of male and female adult stature was examined in Milan (Italy) spanning 2000 years. Interestingly, statistical analyses showed that the simplest model to describe data was based solely on sex and other variables (including historical period) were not able to ameliorate the statistical description, if considered. Notably, no significant changes in stature over time for both sexes were observed. This is, to the best of our knowledge, the first study to show no change in stature trend over time in Europe. Indeed, studies have observed a U-shaped trend in adult stature, with tall individuals in Roman and Early Medieval times, a negative secular trend in the Late Middle Ages and/or Modern era, and a height recovery in the twentieth century^3,8,9,11,13,14,34^. This difference between our study and the cited literature may be explained by several factors. First, the present paper focused on a single place: although it considers individuals from various archaeological sites, all necropolises are in the same geographical area, i.e., the city of Milan. The concentration of the study to such a limited area prevents geographical biases, for instance, arising when considering individuals from both rural and urban areas, or multiple areas with distinct topographies, microclimates, agricultural productions, population densities and economic activities, which would represent different living conditions. In this paper, the geographic and economic context of the study is homogenous and entirely restricted to the urban area of the city of Milan, one of the most important and populous European cities throughout History. Second, in addition to considering individuals from a single geographical context, social and economic disparities were similarly limited. Based on archaeological data, we know that the skeletal remains from the various necropolises selected belonged to individuals from poor social classes. People from middle classes were rare occurrences. Given that socioeconomic factors may impact maximum growth, the fact that the individuals presented a similar socioeconomic background limited variation in the data and reduced biases for their interpretation. Third, the study was performed with the systematic use of a unique methodology. Many methods for the estimation of stature based on regression formulae have been published over the years, including Pearson^35^, Trotter and Gleser^36,37^, Trotter^32^, and Olivier et al*.*^38^ (among others). Although each may be criticized for their reliability with varying degree^39^, Trotter^32^ seems to present small method error, small variation and is still one of the most commonly used methods for stature reconstruction today^13^. These methods were developed from living individuals and as such, the formulae may not be adequate for their application in past populations, as secular change may constitute a bias in the estimation. Yet, regardless of the accuracy of the method in establishing living stature from long bone measurements, all stature estimations were carried out following the same method in the present study, allowing a reliable comparison of the data obtained and hence the analysis of its diachronic trend.

Nonetheless, performing the analysis based on reconstructed statures instead of bone lengths constitutes a limitation of the study, by adding variability to the data sample. In addition, the study assumes a relative homogeneity of the sample, in particular regarding genetics and social status, mainly based on archaeological data, which must be acknowledged.

Koepke and Baten^14,15^ examined a large sample of 2,974 individuals (both male and female) from 314 sites all over Europe, spanning the last 2000 years. After accounting for differences in estimation techniques and separating Europe in various regions (i.e., Central/Western, Estern/Northern and Mediterranean) they observed several trends: specifically, a high stature in the Early Middle Ages followed by a decrease until the thirteenth century, a height recovery in the fourteenth-fifteenth centuries and a second decrease in the seventeenth century, and described an overall stagnant height over the last two millennia. This last result is consistent with our findings, although their sample had, as they acknowledged, low data on Mediterranean countries (which includes Italy), females in general, and individuals after the seventeenth century. The variation they noted over the centuries, which they attributed to a low density of population and urbanization following invasions and plagues (in particular the Justinian plague^16^), warm/cold climate and rapid urbanization, was not observed in our study.

Based on the surprising stability of the data, it can be hypothesized that living conditions in Milan, even for the population belonging to the lower social and economic strata of society, were better than those of other urban areas. Historians have long discussed the survival, but also the continuity and relevance of Milan city life after the Roman age, in spite of the political change and depopulation that followed the institutional end, or rather, the transformation of the Western Roman Empire, and highlighted the presence of mercantile and craft activity, well attested in the centuries of the Early Middle Ages and even more so since the eighth century^40–42^. The city of Saint Ambrosius stood and still stands in a site that has always benefited from natural resources (e.g., water, land, forests, including timber, game, edible fruits and branches to feed animals, and easy supply of metals), which is why since the Carolingian age, the great royal fiscal *curtes*^41^, that is, the assets of kings and queens, which was the material basis and economic support of political power, arose precisely there. In addition to the possibility of transporting and/or trading products, its position along the rivers guaranteed the availability of food resources, thanks to the particular fertility of the land and the richness of the water, which also allowed the irrigation of the lands, and encompassed vast and dense woodlands. In the praising eighth century poem, the *Versum de Mediolano civitate*, Milan is described as "famous for merchandise of all qualities and full of grains of all kinds; there is an abundance of wine, and meat is in large quantities". The poem also tells how the poor received assistance in the city: "the naked are abundantly clothed there; the poor and the Romans are satisfied"^43,44^. Indeed, at least from the Early Middle Ages, Milan hosted various healthcare institutions, including the *xenodochia*, which later became hospitals, dedicated to the assistance of the needy.

Milan in the Middle Ages, despite what has been written about it, clearly differs from the surrounding countryside, even though it is no longer the glorious city of the Roman times, and is characterized by a diverse population in terms of social articulation. A metropolis such as Milan offered chances of social affirmation. The political centrality of the city over the centuries generated the presence of an almost continuous private and public charity, which acted as a true rampart against poverty, and therefore guaranteed a better standard of living for its inhabitants, including the poor^43^. This support offered by the city to the needy may have played a relevant role on the trend of stature.

Centuries later, at the end of the thirteenth century, a text praising the city similar in typology to the above-mentioned *Versum*, entitled *De magnalibus Mediolani* and written by a religious, Bonvesin da la Riva, exalted all the riches of Milan: the abundance of waters, the mildness of the climate, the number of workers and artisans, meadows, fruits, wheat and grains, vines, woods and forests, and the “abundance of foodstuffs”^45^. Among the “greatnesses” of the city, ten urban hospitals can be counted (15 in the surrounding countryside) for the “poor and sick”, which demonstrates how many were cared for in institutions as well as at home. All poor and sick, and even children in need, were granted "with humanity and generosity" the comfort of a bed and food as well as surgical care: "No indigent is rejected here"^45^.

After his conquest of the city in 1450, Francesco Sforza, then Duke of Milan, promoted a series of architectural and infrastructural improvements for the city, including the foundation of a *hospitale magnum*. The *Ospedale Maggiore* was conceived as a reform of the Milanese healthcare system, headquarters for the centralized management of all city hospitals then in existence. Dedicated to the care of the poor, it became a model of healthcare innovation and scientific activity across Europe in the sixteenth and seventeenth centuries. The hospital developed a model of care oriented toward “therapy”, aimed at the rehabilitation and recovery of a workforce that needed to be reinserted into the labor market and its "removal" of the incurable^25^. The patients of the hospital, all admitted based on a *fede di povertà* (“faith of poverty”) were given the comfort of a bed as well as complete meals including bread legumes, wine and occasionally fish, meat, and dairy products^17^. In this sense, the hospital became an actual “factory of health” as well as a large economic company, thanks to its substantial land holdings.

In the modern and contemporary age, the assistance and shelter provided by the *Ospedale Maggiore* proved to be fundamental in warding off delinquency and revolts of the poorer population, since it provided precisely the means of livelihood and care for the thousands of people who turned to it, including abandoned mothers and children. These numbered in the thousands, mostly females (called "Colombe" and taken in at St. Celsus Hospital) and were guaranteed a more than decent standard of living. Despite various attempts at changing welfare policy between the seventeenth and nineteenth centuries that led to problems of various kinds (i.e., runaways and revolts), the "Colombe" continued to enjoy a privileged status and to see the *Ospedale Maggiore* as the institution that would protect them for life, as a true family^46^.

The city's location in a land rich in natural resources, availability of food resources, political power, possibility of defense of the population within the walls, and especially, and more and more as the centuries went by, care of the poor may therefore explain, at least in part, the “stability” of the trend in stature over the centuries, as people were known to leave the countryside and other areas to have a chance at a better life in Milan.

One might speculate, but not yet having confirmation of this due to the scarcity of studies that can be compared, that the genetic input that arose from the mixing for centuries of men and women of different backgrounds contributed to the "physical endurance" of the Milanese population. In theory, the various foreign invasions and occupations of the city of Milan could have led to considerable gene flow, which may have helped maintain stable stature trends despite social and political changes and prevented negative secular trends. However, it should be kept in mind that barbarian migrations always involved a few thousand people, such as the Huns, the Ostrogoths or the Lombards, who settled over vast territorial areas which may have been already densely populated, such as Milan, and so their genetic input could have turned out to be scarce^47^. Furthermore, Wenskus^48^ already argued that the protagonists of the migrations were groups of warriors who did not share specific biological characteristics.

The analysis of other stress markers (such as cribriotic lesions, porotic hyperostosis, enamel hypoplasia and Harris lines) on a sample 200 individuals (50 per historical period, including Roman era, Middle Ages, Modern and Contemporary eras), equally divided between males and females, shows that frequencies of non-mechanical stress were more or less stable in Milan until the Contemporary era, when they dropped notably (from 67 to 26% of individuals)^49^. These results strengthen those of the present study, showing a concordant trend between stature and other stress markers, until the nineteenth century, when they diverge. This divergence may be due to the sensitivity of stature to non-mechanical stressors, which may be different from the other stress markers previously mentioned.

To further our understanding of health and stature variation in the past, one perspective would be to perform paleogenomic analyses on the skeletal remains of the study sample in order to confront the “potential” stature of the individuals, genetically determined and obtained from paleogenomic data, to that actually reached and osteologically calculated from the skeletal remains^2^. This way, we may be able to distinguish between individuals who reached their full genetic growth potential and those who did not, and better define the evolutionary trend of stature variation over time and the events and conditions that influenced it.

## Conclusion

The present paper describes the results of the first study on the stature of the Milanese population over a time interval of about 2000 years. This comes as part of a larger project aimed at reconstructing the city of Milan throughout time and better understanding the history of its inhabitants, and in particular those often forgotten and neglected by History, such as the poor masses of the common people.

As a result of the study, we observed that the diachronic trend of mean stature in both males and females remained stable over time and statistical analyses showed that historical periods did not significantly influence the variation of the data. Since all samples were processed according to the same methods, there is no reason to assume a source of discontinuity between the taken measurements. The statistical results must therefore depend on other factors, not necessarily isolated from each other. The most reasonable is the fact that by drawing exclusively from the Milan urban area, the variability of the studied sample would be more limited. We propose that the stable trend of stature over time may be related to the relatively better living conditions in the city of Milan, with respect to other areas.

## Supplementary Information

Supplementary Information.

## Data Availability

All data generated and analyzed during this study are included in this published article and its supplementary information file.
